# Supplementary material for: Tongue swab Mycobacterium tuberculosis qPCR for community screening of asymptomatic TB vs. clinic-based triage of symptomatic TB
Source: medRxiv. 2026 Feb 14:2026.02.12.26346160. Preprint. [Version 1] doi: 10.64898/2026.02.12.26346160 (PMC12919145; doi:10.64898/2026.02.12.26346160)

Supplementary Material

Tongue swab *Mycobacterium tuberculosis* qPCR for community screening of asymptomatic TB vs. clinic-based triage of symptomatic TB

Rachel C. Wood[1], Alaina M. Olson[1], Katherine A. Lochner[2], Rane B. Dragovich[1], Alexey Ball[3], Amy Steadman[3], Tahlia Perumal[4,5], Simon C. Mendelsohn[6], Humphrey Mulenga[6], Michele Tameris[6], Denis Awany[6], Tumelo Moloantoa[7], Stephanus T. Malherbe[8], Austin Katona[9], Fernanda Maruri[9], Kris M. Weigel[1], Firdows Noor[8], Ravindre Panchia[7], Khuthadzo Hlongwane[7], Kim Stanley[8], Yuri F. van der Heijden[9,10], Katie Hadley[6], Gerhard Walzl[8], Thomas J. Scriba[6], Neil A. Martinson[7], Keertan Dheda[4], Al Leslie[11], Bernard Fourie[12], Timothy R. Sterling[9], Gerard A. Cangelosi[1*], Mark Hatherill[6*] and the RePORT South Africa Study Team

[1]University of Washington, Department of Occupational & Environmental Health Sciences, Seattle, Washington, USA
[2]Johns Hopkins University Center for TB Research, Baltimore, Maryland, USA
[3]Global Health Labs, Bellevue, Washington, USA
[4]University of Cape Town Lung Institute, Centre for Lung Infection and Immunity, Cape Town, South Africa
[5]South African Medical Research Council/University of Cape Town, Centre for the Study of Antimicrobial Resistance, Cape Town, South Africa
[6]South African Tuberculosis Vaccine Initiative, Institute of Infectious Disease and Molecular Medicine and Department of Pathology, University of Cape Town, Cape Town, South Africa
[7]Perinatal HIV Research Unit (PHRU), University of the Witwatersrand, Johannesburg, South Africa
[8]South African Medical Research Council Centre for TB Research; Division of Immunology, Department of Biomedical Sciences, Faculty of Medicine and Health Sciences, Stellenbosch University, Cape Town, South Africa
[9]Vanderbilt Tuberculosis Center, Vanderbilt University Medical Center, Nashville, Tennessee, USA
[10]The Aurum Institute, Johannesburg, South Africa
[11]Africa Health Research Institute, Somkhele, Mtubatuba, South Africa
[12]Tuberculosis Research Institute, University of Pretoria, Pretoria, South Africa

*Contributed equally

Corresponding Author: Professor Mark Hatherill, South African Tuberculosis Vaccine Initiative, Institute of Infectious Disease and Molecular Medicine and Division of Immunology, Department of Pathology, University of Cape Town, Anzio Road, Observatory, 7925, South Africa.
Email mark.hatherill@uct.ac.za; Telephone +27214066791.

Table of Contents

Pages 3-5: **Supplemental Methods**

Pages 6-7: **Table S1.** List of RePORT South Africa study team members

Pages 8-9: **Table S2.** Demographic and clinical characteristics of participants in the Clinic Cohort, differentiated by testing method

Pages 10: **Table S3.** Sensitivity and specificity of tongue swab qPCR in the Clinic and HHC Cohorts

Page 11: **Figure S1.** Tongue swab, sputum culture and smear, chest radiograph, and symptom positivity by sputum Xpert Ultra grade among (A) symptomatic and (B) asymptomatic participants

Page 12: **Figure S2.** Proportion of sputum Xpert Ultra semi-quantitative grades in TB Cases by cohort.

Supplemental Methods

Household Contact Cohort samples

Household Contact (HHC) Cohort samples were tested at Global Health Labs (Bellevue, WA, USA) by RCW, KAL, and AB. In the published protocol (1), tongue swabs (Regular FLOQSwabs®, 520CS01, Copan Italia, Brescia, Italy) were collected from participants and immediately placed in tubes containing 500 µL 1x Tris-EDTA (TE) buffer and tested the same day. In this study, the dry tongue swabs were removed from the -80 °C freezer and heated at 100 °C for 10 minutes to achieve MTB sterilization, 500 µL TE buffer was then added, and samples were vortexed for 15 seconds at high speed. Samples were then incubated for a further 10 minutes at 100 °C and tested as previously described in Steadman *et al.*, 2024 (1). For the qPCR analysis, 250 µL of the sample was divided into 5 reaction wells, each containing 50 µL to maximize the amount of sample analyzed. One positive control (healthy volunteer tongue swab spiked with an estimated 10,000 *Mycobacterium tuberculosis* H37Ra cells (cultured and stored frozen at pre-determined concentrations) and one negative control (500 µL of TE buffer) were processed and tested alongside every 14 clinical samples. A 10-fold dilution standard curve (0.1 to 100,000 genomes per reaction) using quantitative genomic *Mycobacterium tuberculosis* H37Rv DNA (ATCC 25618DQ, Manassas, Virginia, USA) diluted in molecular grade water was also plated in duplicate. Detection of amplification, with a Cq cut-off threshold of 40 and a fluorescence export threshold of 100,000 Δ Rn for the IS*6110* target sequence, in any of the 5 replicate wells was considered a positive test result. Results for IS*1081* were also obtained using a different fluorescent probe in the multiplex qPCR assay.

Clinic Cohort Samples

High-volume qPCR

All Clinic Cohort samples were tested at the University of Washington (Seattle, WA, USA). Both the HHC Cohort samples and half of the Clinic Cohort samples were tested using the high-volume qPCR method; however, there were some lab-specific instrumentation differences for the Clinic Cohort: a Fisher Scientific Isotemp heat block (88-860-023, Waltham, MA, USA) rather than a Thermo Scientific heat block (88-870-006, Waltham, MA, USA); a CFX96 Deep Well thermocycler (Bio-Rad 3600037, Hercules, CA, USA) rather than a QuantStudio 5 (Applied Biosystems A34322, Waltham, MA, USA) (qPCR cycling parameters were sustained); and fluorescence thresholds set to 500 RFU (Bio-Rad) versus 100,000 Δ Rn (QuantStudio 5), as described above. The high-volume qPCR testing of the Clinic Cohort samples was done by RBD and RCW.

SSMaC with qPCR

The remaining Clinic Cohort samples were tested by SSMaC with qPCR, IS*6110*- and IS*1081*-targeted probe-based DNA capture and qPCR by AMO and RBD (2). Unlike the previously described protocol, which used archived lysates, sample processing began from dry, frozen (-80 °C) tongue swabs. Samples were transferred from -80 °C to an incubation at 100 °C for 10 minutes, supplemented with 500 µL of 1x TE, vortexed for 15 seconds at maximum speed, and further incubated at 100 °C for 10 minutes. Premeasured 150 mg of 0.1 mm glass disruption beads (Research Products International 9830, Mt. Prospect, IL, USA) were added to each tube, and samples were bead-beaten horizontally for 20 minutes at maximum speed on a vortex adapter (QIAGEN, 13000-V1-24, Hilden, Germany). Lysates were transferred to 1.5-mL LoBind tubes

and SSMaC and qPCR proceeded per Olson *et al.*, 2025 (2), including positive and negative extraction controls. The export threshold for the combined FAM channel (targeting IS*6110* and IS*1081*) used in this analysis was 2,250 Δ Rn. If a negative extraction control amplified (<40 Cq), the entire sample set was rerun from reserved eluate with fresh qPCR reagents after decontaminating workspaces and equipment. Sets in which the contamination issue resolved on rerun were retained for analyses; those that did not were excluded.

References

1. Steadman A, Andama A, Ball A, Mukwatamundu J, Khimani K, Mochizuki T, et al. New Manual Quantitative Polymerase Chain Reaction Assay Validated on Tongue Swabs Collected and Processed in Uganda Shows Sensitivity That Rivals Sputum-based Molecular Tuberculosis Diagnostics. Clin Infect Dis. 2024;78(5):1313-20.
2. Olson AM, Wood RC, Weigel KM, Yan AJ, Lochner KA, Dragovich RB, et al. High-sensitivity detection of *Mycobacterium tuberculosis* DNA in tongue swab samples. Journal of Clinical Microbiology. 2025;63(2):e01140-24

Supplemental Tables and Figures

Table S1. List of RePORT South Africa study team members.

Name	Affiliation
Pattamukkil Abraham	Perinatal HIV Research Unit, University of the Witwatersrand
Thakiera Allie	South African Tuberculosis Vaccine Initiative, University of Cape Town
Cynthia Baard	Department of Paediatrics and Child Health, University of Cape Town
Zainab Baig	Africa Health Research Institute
John Belisle	Colorado State University
Nicole Bilek	South African Tuberculosis Vaccine Initiative, University of Cape Town
ACE Carstens	Department of Biomedical Sciences, Stellenbosch University
Novel N. Chegou	Department of Biomedical Sciences, Stellenbosch University
Kevyna Chetty	Africa Health Research Institute
Yolundi Cloete	South African Tuberculosis Vaccine Initiative, University of Cape Town
Marwou de Kock	South African Tuberculosis Vaccine Initiative, University of Cape Town
Gareta Dickman	Africa Health Research Institute
Charity Dire	Perinatal HIV Research Unit, University of the Witwatersrand
Karen Dobos	Colorado State University
Stephany Norah Duda	Vanderbilt Tuberculosis Center, Vanderbilt University Medical Center
Mzwandile Erasmus	South African Tuberculosis Vaccine Initiative, University of Cape Town
Marina Cruvinel Figueiredo	Vanderbilt Tuberculosis Center, Vanderbilt University Medical Center
Marika Flinn	Department of Biomedical Sciences, Stellenbosch University
Travis Harris	Vanderbilt Tuberculosis Center, Vanderbilt University Medical Center
Andriette Hiemstra	Department of Biomedical Sciences, Stellenbosch University
Shameem Jaumdally	University of Cape Town Lung Institute
Ryan Johnson	University of Cape Town
Farina Karim	Africa Health Research Institute
Masooda Kaskar	South African Tuberculosis Vaccine Initiative, University of Cape Town
Nobulumko Khomba	South African Tuberculosis Vaccine Initiative, University of Cape Town
Thandeka Khoza	Africa Health Research Institute
Léanie Kleynhans	Department of Biomedical Sciences, Stellenbosch University; Mater Research Institute – The University of Queensland
Tahira Kootbodien	University of Cape Town
Andrea Kotze	University of Cape Town Lung Institute
Belinda A Kriel	Department of Biomedical Sciences, Stellenbosch University
Ané Kruger	Department of Biomedical Sciences, Stellenbosch University
Lorraine Lichakane	Perinatal HIV Research Unit, University of the Witwatersrand
Ilze Louw	Department of Biomedical Sciences, Stellenbosch University
Angelique Luabeya	South African Tuberculosis Vaccine Initiative, University of Cape Town
Candice MacDonald	Department of Biomedical Sciences, Stellenbosch University
Lindiwe Madziwa	Africa Health Research Institute
Lebohang Makhethe	South African Tuberculosis Vaccine Initiative, University of Cape Town
Sandisiwe Mangali	South African Tuberculosis Vaccine Initiative, University of Cape Town
Linda Mbuthini	University of Cape Town

Carolina Mehaffy	Colorado State University
Thabang Moloja	Perinatal HIV Research Unit, University of the Witwatersrand
Angelique Mouton	South African Tuberculosis Vaccine Initiative, University of Cape Town
Evans Muchiri	South African Tuberculosis Vaccine Initiative, University of Cape Town
Mbusiseni Ngema	Perinatal HIV Research Unit, University of the Witwatersrand
Hlengiwe Nkambule	South African Tuberculosis Vaccine Initiative, University of Cape Town
Onke Nombida	South African Tuberculosis Vaccine Initiative, University of Cape Town
Sarah Nyangu	South African Tuberculosis Vaccine Initiative, University of Cape Town
Fajwa Opperman	South African Tuberculosis Vaccine Initiative, University of Cape Town
Gregory Ording-Jespersen	Africa Health Research Institute
Kennedy Otwombe	Perinatal HIV Research Unit, University of the Witwatersrand
Will Ramsay	Vanderbilt Tuberculosis Center, Vanderbilt University Medical Center
Tracy Richardson	Department of Biomedical Sciences, Stellenbosch University
Carmen Segelaar	South African Tuberculosis Vaccine Initiative, University of Cape Town
Jane Shaw	Department of Biomedical Sciences, Stellenbosch University
Kimberly Shelton	Colorado State University
Justin Shenje	South African Tuberculosis Vaccine Initiative, University of Cape Town
Theresa Smit	Africa Health Research Institute
Bronwyn Smith	Department of Biomedical Sciences, Stellenbosch University
Candice Snyders	Department of Biomedical Sciences, Stellenbosch University
Marcia Steyn	South African Tuberculosis Vaccine Initiative, University of Cape Town
Sara Suliman	University of California San Francisco
Floris Swanepoel	Perinatal HIV Research Unit, University of the Witwatersrand
Lorraine Thobakgale	Perinatal HIV Research Unit, University of the Witwatersrand
Susanne Tonsing	Department of Biomedical Sciences, Stellenbosch University
Nicolette Tredoux	South African Tuberculosis Vaccine Initiative, University of Cape Town
Megan Turner	Vanderbilt Tuberculosis Center, Vanderbilt University Medical Center
Petrus Tyambetyu	South African Tuberculosis Vaccine Initiative, University of Cape Town
Habibullah Valley	South African Tuberculosis Vaccine Initiative, University of Cape Town
Lyle van de Berg	University of Pretoria
Yuri van der Heijden	Vanderbilt Tuberculosis Center, Vanderbilt University Medical Center
Gian van der Spuy	Department of Biomedical Sciences, Stellenbosch University
Ilana R van Rensburg	Department of Biomedical Sciences, Stellenbosch University
Johanna E van Rooyen	South African Tuberculosis Vaccine Initiative, University of Cape Town
Hilary Vansell Riley	Vanderbilt Tuberculosis Center, Vanderbilt University Medical Center
Ashley Veldsman	South African Tuberculosis Vaccine Initiative, University of Cape Town
Lindsay Wilson	University of Cape Town
Lesley Workman	Department of Paediatrics and Child Health, University of Cape Town
Heather Zar	Department of Paediatrics and Child Health, University of Cape Town

Table S2. Demographic and clinical characteristics of participants in the Clinic Cohort, differentiated by testing method.

	**Total participants (N = 673)**	**High-volume Method (N = 334)**	**SSMaC Method (N = 339)**	***p*-value (High-volume vs SSMaC)**
	**n (%)**	**n (%)**	**n (%)**	
Site				
AHRI	312 (46.4)	153 (45.8)	159 (46.9)	0.77
PHRU	28 (4.2)	16 (4.8)	12 (3.5)	0.40
SUN	103 (15.3)	47 (14.1)	56 (16.5)	0.39
UCT-LI	172 (25.6)	85 (25.4)	87 (25.7)	0.93
UP	58 (8.6)	33 (9.9)	25 (7.4)	0.25
Age, years (median, IQR)	36 (28–44)	36 (27–44)	36 (28–45)	N/A
Sex (female)	245 (36.4)	115 (34.4)	130 (38.3)	0.29
HIV Positive	204 (30.3)	99 (29.6)	105 (31.0)	0.69
Any symptom	673 (100)	334 (100)	339 (100)	0.99
Cough	588 (87.4)	293 (87.7)	295 (87.0)	0.79
Fever	215 (31.9)	108 (32.3)	107 (31.6)	0.85
Weight loss	365 (54.2)	191 (57.2)	174 (51.3)	0.12
Fatigue	323 (48.0)	163 (48.8)	160 (47.2)	0.67
Night sweats	370 (55.0)	190 (56.9)	180 (53.1)	0.32
Chest pains	258 (38.3)	140 (41.9)	118 (34.8)	0.06
Smoking				
Unknown	1	0	1	0.32
Current	299/672 (44.4)	157 (47.0)	142/338 (42.0)	0.19
Former	77/672 (11.4)	34 (10.2)	43/338 (12.7)	0.31
Never	296/672 (44.0)	143 (42.8)	153/338 (45.3)	0.52
Prior TB	148 (22.0)	73 (21.9)	75 (22.1)	0.95
Chest radiograph				
Unknown	332	167	165	0.73
Normal	176/341 (51.6)	86/167 (51.5)	90/174 (51.7)	0.97
Abnormal	165/341 (48.4)	81/167 (48.5)	84/174 (48.3)	0.97
Positive sputum smear microscopy	165 (24.5)	79 (23.7)	86 (25.4)	0.61
Not done	4	2	2	1.0
3+ grade	45/165 (27.3)	17/79 (21.5)	28/86 (32.6)	0.11
2+ grade	53/165 (32.1)	31/79 (39.2)	22/86 (25.6)	0.06
1+ grade	38/165 (23.0)	17/79 (21.5)	21/86 (24.4)	0.66
Scanty grade	28/165 (17.0)	14/79 (17.7)	14/86 (16.3)	0.81
Positive sputum culture	206/672 (30.7)	100/330 (30.3)	106/338 (31.4)	0.76

Not done	5	4	1	0.18
MGIT time to positivity, days (Median, IQR)	9 (6–14)	10 (6–15)	9 (6–14)	N/A
Positive sputum Xpert Ultra	221/669 (33.0)	110 (32.9)	111/335 (33.1)	0.95
Not done	4	0	4	0.04*
High SQ grade	77/221 (34.8)	33/110 (30.0)	44/111 (39.6)	0.13
Medium SQ grade	42/221 (19.0)	21/110 (19.1)	21/111 (18.9)	0.97
Low SQ grade	56/221 (25.3)	31/110 (28.2)	25/111 (22.5)	0.33
Very low SQ grade	26/221 (11.8)	15/110 (13.6)	11/111 (9.9)	0.40
Trace SQ grade	19/221 (8.6)	10/110 (9.1)	9/111 (8.1)	0.79
SQ grade not recorded	1/221 (0.5)	0/110 (0.0)	1/111 (0.9)	0.32

AHRI, Africa Health Research Institute. **PHRU**, Perinatal HIV Research Unit, University of the Witwatersrand. **SUN**, Stellenbosch University. **UCT-LI**, University of Cape Town-Lung Institute. **UP,** University of Pretoria. SSMaC, sequence-specific magnetic capture. HHC, household contact. SQ, semi-quantitative. IQR, inter-quartile range. *$p < 0.05$.

Table S3. Sensitivity and specificity of tongue swab qPCR in the Clinic and HHC Cohorts; results from **A.** only regular Copan flocked swabs, and **B**. only swabs that are not regular Copan flocked swabs. The *p*-values show whether the sensitivity or specificity of the cohort/method is significant relative to the overall sensitivity or specificity. **p*-value is significant at $p < 0.05$ by 2-population proportion Z-test. SSMaC, sequence-specific magnetic capture. HHC, household contact.

A.

	**Sensitivity**			**Specificity**		
	**n/N**	**% (95% CI)**	***p*-value**	**n/N**	**% (95% CI)**	***p*-value**
**Clinic Cohort**						
High-volume qPCR	65/102	63.7% (53.6–73.0)	0.99	188/200	94.0% (89.8–96.9)	0.87
SSMaC with qPCR	79/107	73.8% (64.5–81.9)	0.92	192/204	94.1% (90.0–96.9)	0.83
**HHC Cohort**						
High-volume qPCR	8/27	29.6% (13.8–50.2)	0.70	103/106	97.2% (92.0–99.4)	0.08

B.

	**Sensitivity**			**Specificity**		
	**n/N**	**% (95% CI)**	***p*-value**	**n/N**	**% (95% CI)**	***p*-value**
**Clinic Cohort**						
High-volume qPCR	2/3	66.7% (9.4–99.2)	0.92	13/13	100% (75.3–100)	0.38
SSMaC with qPCR	3/5	60.0% (14.7–94.7)	0.52	19/19	100% (82.4-100)	0.30
**HHC Cohort**						
High-volume qPCR	7/17	41.2% (18.4–67.1)	0.60	22/30	73.3% (54.1–87.7	0.004*

Figure S1. Tongue swab, sputum culture and smear, chest radiograph, and symptom positivity (%) by sputum Xpert Ultra grade among **(A)** symptomatic and **(B)** asymptomatic participants. The numbers within the bars are the test result N. HHC, household contact. TS, tongue swab. CXR, chest radiograph.

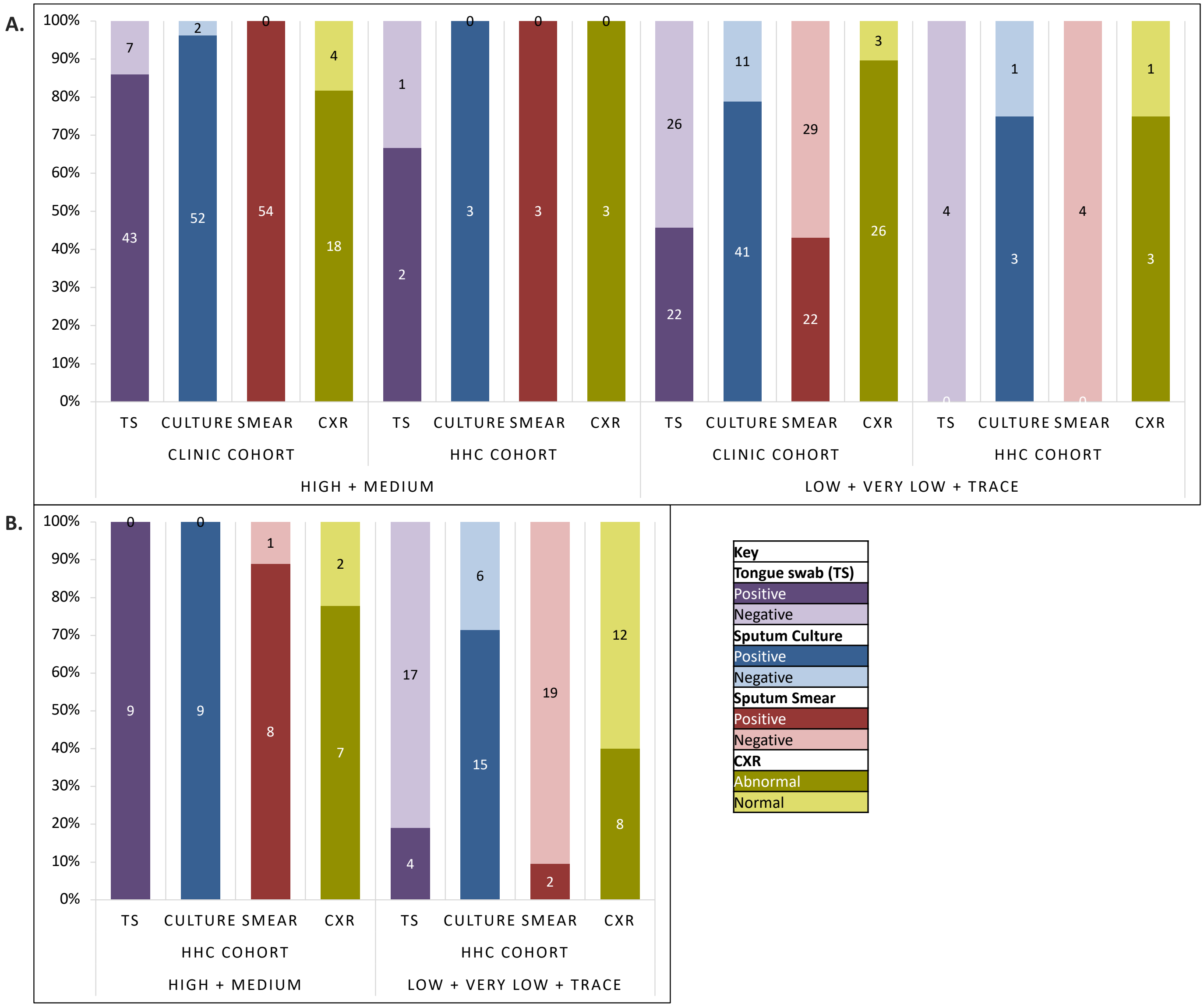

Figure S2. Proportion of sputum Xpert Ultra semi-quantitative grades in TB Cases by cohort. Sputum Xpert Ultra semi-quantitative results are provided as a proxy for MTB bacillary load. Cx, culture. HHC, household contact.

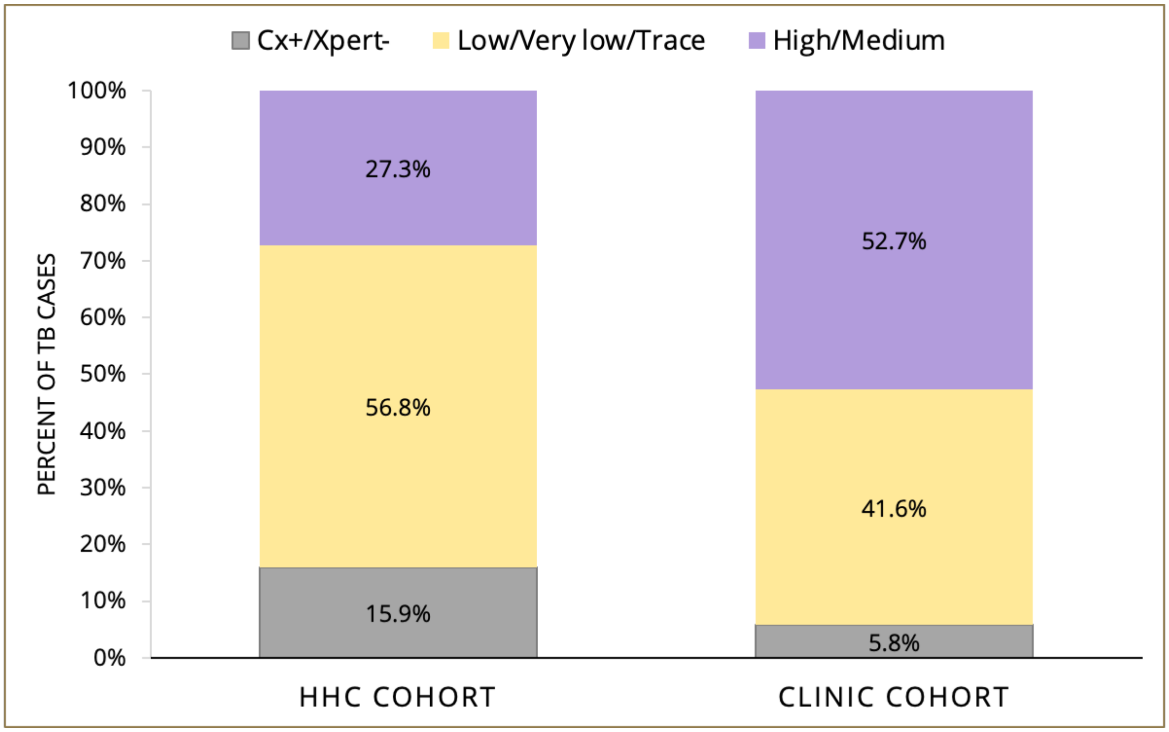

Supplement: Supplement 1 [file media-1.pdf]
